# Drug regimens identified and optimized by output-driven platform markedly reduce tuberculosis treatment time

**DOI:** 10.1038/ncomms14183

**Published:** 2017-01-24

**Authors:** Bai-Yu Lee, Daniel L. Clemens, Aleidy Silva, Barbara Jane Dillon, Saša Masleša-Galić, Susana Nava, Xianting Ding, Chih-Ming Ho, Marcus A. Horwitz

**Affiliations:** 1 Division of Infectious Diseases, Department of Medicine, University of California, Los Angeles, California 90095, USA; 2 Department of Mechanical and Aerospace Engineering, University of California, Los Angeles, California 90095, USA; 3 Med-X Research Institute, School of Biomedical Engineering, Shanghai Jiao Tong University, Shanghai 200030, China; 4 Department of Bioengineering, University of California, Los Angeles, California 90095, USA

## Abstract

The current drug regimens for treating tuberculosis are lengthy and onerous, and hence complicated by poor adherence leading to drug resistance and disease relapse. Previously, using an output-driven optimization platform and an *in vitro* macrophage model of *Mycobacterium tuberculosis* infection, we identified several experimental drug regimens among billions of possible drug-dose combinations that outperform the current standard regimen. Here we use this platform to optimize the *in vivo* drug doses of two of these regimens in a mouse model of pulmonary tuberculosis. The experimental regimens kill *M. tuberculosis* much more rapidly than the standard regimen and reduce treatment time to relapse-free cure by 75%. Thus, these regimens have the potential to provide a markedly shorter course of treatment for tuberculosis in humans. As these regimens omit isoniazid, rifampicin, fluoroquinolones and injectable aminoglycosides, they would be suitable for treating many cases of multidrug and extensively drug-resistant tuberculosis.

## Abstract (web summary)

Current antibiotic therapies for tuberculosis are lengthy and onerous. Here, the authors use an output-driven approach to optimize drug doses for two experimental drug regimens in a mouse model of tuberculosis, leading to improved regimens that reduce treatment time by 75%.

Tuberculosis (TB) is a major health problem of global proportions. In 2014, an estimated 9.6 million people fell ill with TB and 1.5 million people died, placing TB above HIV as the number one infectious cause of death worldwide^1^. Although drug-sensitive TB, which comprises 96.7% of new cases, is a treatable disease, the current standard treatment requires 6–8 months of a multi-drug regimen to achieve relapse-free cure^2^,^3^. This long course of treatment is often associated with toxicity, poor adherence and development of drug resistance. A more effective drug combination that provides more rapid sterilization of tissues has the potential to ameliorate these critical problems^4^,^5^,^6^.

History suggests that the long duration of treatment required to achieve relapse-free cure may be attributable more to limitations in bactericidal activity of the antimicrobial drug combination than to the intrinsic biology of *Mycobacterium tuberculosis*. The first regimen in 1952, consisting of streptomycin, isoniazid (INH) and para-aminosalicylic acid required 24 months therapy; replacing para-aminosalicylic acid by ethambutol (EMB) in the 1960s and subsequently adding rifampicin (RIF) in the 1970s sequentially shortened treatment duration first to 18 months and then to 9–12 months, and substituting pyrazinamide (PZA) for streptomycin in the 1980s further cut treatment duration to 6–8 months^7^.

These previous treatment regimens, culminating in today's Standard Regimen (INH, RIF, EMB and PZA), in which one drug was added or substituted at a time, were not based on a systematic search to identify the most highly synergistic drug combinations. Indeed, contrary to the concept of synergy, two of the four drugs in the Standard Regimen (EMB and PZA) are dropped after 2 months to reduce patient exposure to potential toxic side effects, as by this point they are considered superfluous to treatment and no longer needed to prevent the emergence of drug resistance. Thus, it is likely that an approach centred on identifying the most highly synergistic drug combinations has the potential to shorten further the duration of treatment required to achieve relapse-free cure of TB.

Since conventional evaluation of a large number of TB drugs, even at a few doses, would require testing of an enormous number of drug-dose combinations, a systematic approach is required to identify the most promising drug combinations for treating TB. We have previously developed a method, called Feedback System Control, Second Generation (FSC.II) or Parabolic Response Surface (PRS), in which experimental data is used to solve the second order algebraic equation that describes the drug dose-efficacy response of a multi-drug combination. The PRS approach can map out the entire drug dose-efficacy landscape from a very large search space and rapidly home in on optimal drug-dose combinations. In previous work, PRS enabled us to identify numerous combinations of TB drugs that exhibit higher antimicrobial activity than the Standard Regimen (INH, RIF, EMB and PZA) *in vitro* in a human macrophage model of *M. tuberculosis* infection^8^. Both fluorescence-based bacterial metabolic activity and confirmatory CFU-based killing studies identified two regimens as having greater efficacy than the Standard Regimen—PRS Regimen I (Clofazimine (CLZ)/EMB/Prothionamide (PRO)/PZA) and PRS Regimen II (CLZ/EMB/Bedaquiline (BDQ)/PZA), in which PRO was replaced with the recently developed drug, BDQ. However, preclinical studies in animal models are essential to evaluating the safety and efficacy of new multidrug regimens and determining which combinations merit advancement to clinical trials^9^. While no animal model is perfect, the BALB/c mouse model of pulmonary TB has been broadly predictive of human clinical results and provides a practicable model for comparing multiple TB drug regimens^10^,^11^,^12^—such as those that we identified using the PRS method *in vitro*.

Here, using the mouse model, we apply the PRS method to model the parabolic drug dose-efficacy response surface^13^,^14^,^15^,^16^,^17^,^18^,^19^,^20^ of two of the regimens and thereby identify optimal drug doses for use of these regimens *in vivo.* Since *in vivo* and *in vitro* drug dose ratios can differ markedly, dose re-optimization is a crucial step for ensuring the success of *in vivo* studies. As the PRS approach to transitioning from *in vitro* to *in vivo* drug doses is output-driven, it is agnostic to such considerations as drug mechanism and bacterial metabolic state, automatically takes into account drug–drug interactions, and is not dependent on pharmacokinetic studies. We then compare the two optimized experimental regimens with the Standard Regimen for time required to attain culture negativity in infected lung tissues and to achieve relapse-free cure.

## Results

### Efficacy study of PRS Regimen I

**Figure 1:**
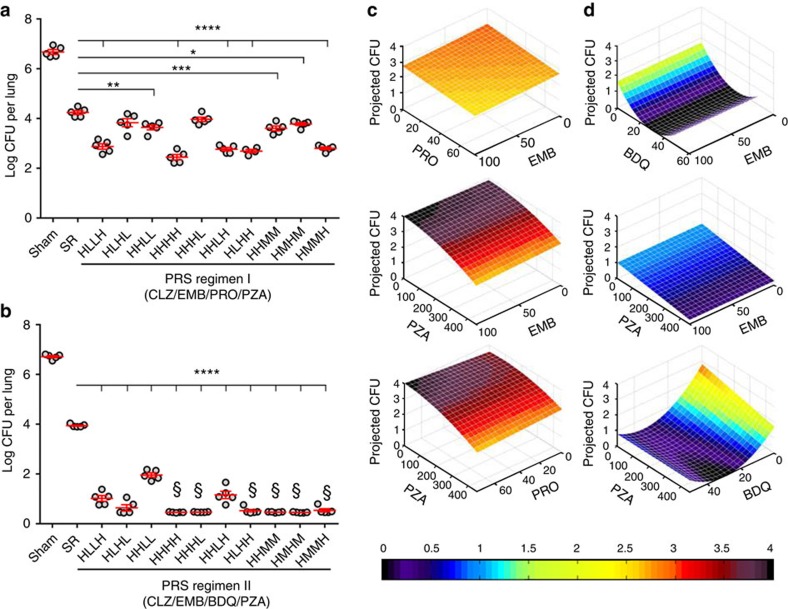
Short-term efficacy studies of PRS Regimens I and II. (**a**,**b**) Lung burden of *M. tuberculosis* in mice that were sham-treated, treated with the Standard Regimen (SR) or treated with PRS Regimens I (**a**) or II (**b**) with the drugs administered at high (H), middle (M) or low (L) dose five times per week for 4 weeks. Data are mean±s.e.m. of log_10_ CFU for *n*=5 mice per group. All treatment groups had significantly fewer CFU than the sham-treated group (*P*<0.0001). Differences in treatment efficacy between the Standard Regimen and individual PRS Regimen I or II groups were evaluated by one-way ANOVA with Tukey's correction. *P<0.05, **P<0.01, ***P<0.001, ****P<0.0001. ^§^No *M. tuberculosis* CFU detected—data plotted as discussed in Methods. (**c**,**d**) Heat maps of the drug-dose response surface for PRS Regimens I (**c**) and II (**d**). These 3-dimensional graphs show how the projected number of lung CFU changes as the dose of one and/or the other drug is increased or decreased. Drug dose is shown in mg kg^−1^. In these plots, in addition to CLZ, the third drug is kept at the high dose.

To determine the optimal dose for individual drugs in PRS Regimen I, we mapped the drug dose-efficacy response surface after a 4-week course of treatment in a mouse model of pulmonary TB. For each drug, the highest dose used was equal to the highest dose previously used in mouse models of pulmonary TB in the literature, and the dose range chosen included the standard human equivalent dose. To reduce the number of different treatment groups required to map the drug dose-efficacy response surface with PRS methodology to a practicable number (10 groups), we held the dose of one (CLZ) of the 4 drugs constant, while varying the doses of the other three drugs. We infected mice by aerosol with the highly virulent *M. tuberculosis* strain Erdman, delivering 2.22±0.09 (mean±s.e.m.) log_10_ colony forming units (CFU) of *M. tuberculosis* per lung. Higher challenge doses, such as those used by others^10^ in similar types of studies employing the less virulent *M. tuberculosis* strain H37Rv (ref. 21), could not be used because mice challenged with such doses of the Erdman strain died before treatment could be initiated at two-weeks post-challenge. By two weeks post challenge, the burden of *M. tuberculosis* had increased by 4 logs to 6.24±0.05 log_10_ CFU per lung, at which point we initiated antibiotic treatment by oral gavage five times a week (Monday—Friday). Ten groups of mice received CLZ at 25 mg kg^−1^ and EMB, PRO and PZA were given at permutations of high, middle (1/3rd the high dose) or low (1/9th the high dose) doses as indicated in Table 1; Fig. 1a. As controls, one group of mice was treated with the Standard Regimen (INH/RIF/EMB/PZA) and another group received sham treatment.

**Figure 2:**
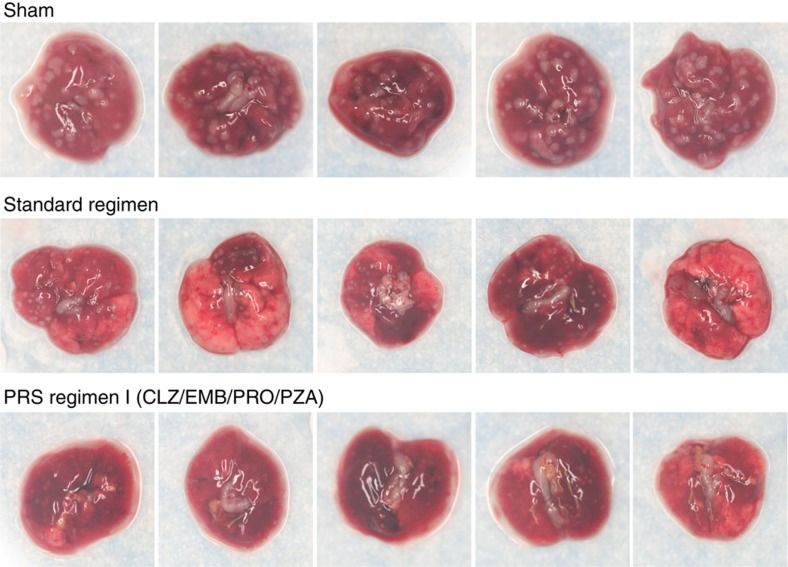
Lung pathology of mice that received sham or Standard Regimen or PRS Regimen I treatment. Shown are representative gross pathology images of lungs dissected from mice that were sham-treated or treated with the Standard Regimen or PRS Regimen I at high doses of each drug by oral gavage five times (Monday—Friday) a week for 4 weeks.

On completion of the 4-week treatment, the sham-treated mice had 6.67±0.09 log_10_ CFU of *M. tuberculosis* in their lungs (Fig. 1a; Supplementary Table 1). Treatment with the Standard Regimen significantly reduced lung burden by 2.43 log_10_ CFU (*P*<0.0001, one-way ANOVA with Tukey's correction). Mice in all 10 PRS Regimen I-treated groups had fewer CFU in their lungs than mice treated with the Standard Regimen. The best PRS Regimen I group (that is HHHH) had 1.79 logs fewer CFU in their lungs than mice treated with the Standard Regimen (*P*<0.0001, one-way ANOVA with Tukey's correction). These results correlated with the severity of gross pathology and number of tubercles on the lung surface (Fig. 2). Thus, PRS Regimen I has markedly greater bactericidal activity in the mouse model than the Standard Regimen. On completion of the 4-week treatment, the sham-treated mice had 4.07±0.19 log_10_ CFU of *M. tuberculosis* in the liver and 5.03±0.11 log_10_ CFU in the spleen, while all treated groups had <2.2 log_10_ CFU in the liver and less than 1.7 log_10_ CFU in the spleen.

A parabolic drug dose-efficacy response surface was generated from the regression equation fitted to the lung log_10_ CFU data from the 10 groups of mice treated with various drug-dose combinations of PRS Regimen I (Methods, equation 2). This drug dose-efficacy response surface had a fitting correlation (*r*) of 0.999 between the measured log_10_ CFU and the projected log_10_ CFU and an adjusted *R*^2^ value of 0.993 indicating that the model explains 99.3% of the variability in the projected log_10_ CFU. A comparison of the observed means versus the predicted means of the response surface model showed a per cent difference {per cent difference=100 × (observed mean—predicted mean)/((observed mean+predicted mean)/2)} of ≤1.4% with an average difference of 0.036%. Levene's test showed no deviation from homoscedasticity among the 10 PRS Regimen I s.d., satisfying the requirements for s.d. pooling in the parabolic and ANOVA analyses.

We found that, with CLZ and PZA doses kept constant, the drug dose-response surface for EMB and PRO was relatively flat, as dose variations of these two drugs had relatively little influence on the efficacy of PRS Regimen I to reduce the lung burden of *M. tuberculosis* (Fig. 1c, top). In contrast, differences in PZA dose had a major impact on lung burden reduction of the combination regimen (Fig. 1c, middle and bottom). Among the 10 PRS Regimen I-treated groups, mice treated with the individual drugs at their highest dose had the lowest lung burden of *M. tuberculosis*, whereas mice treated with the identical drug doses except for PZA, which was at low dose, had the highest lung bacterial burden. The difference in bacterial burden in the lungs between these two groups was highly significant (*P*<0.0001, one-way ANOVA with Tukey's correction). Thus, both the drugs and the drug ratio in a drug combination are important to the outcome of the treatment.

### Efficacy study of PRS Regimen II

**Figure 3:**
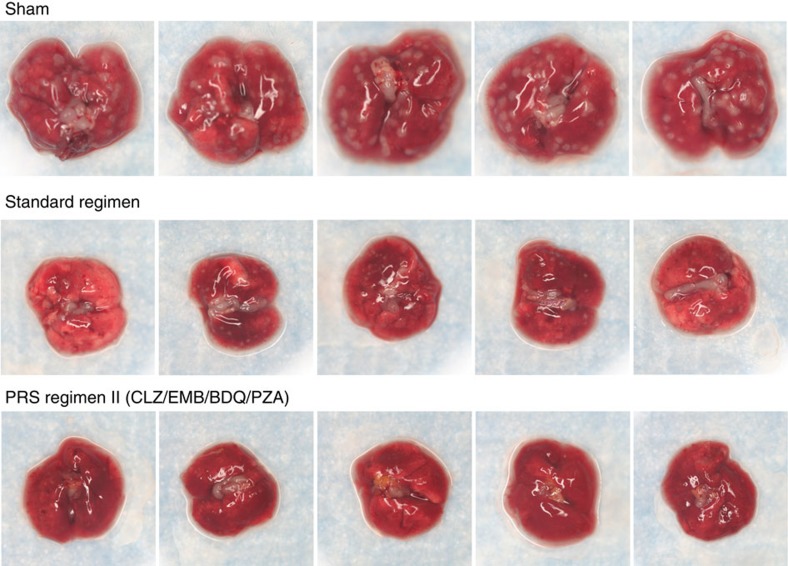
Lung pathology of mice that received sham or Standard Regimen or PRS Regimen II treatment. Shown are representative gross pathology images of lungs dissected from mice that were sham-treated or treated with the Standard Regimen or PRS Regimen II at high doses of each drug by oral gavage five times (Monday—Friday) a week for 4 weeks.

We applied the same strategy to map the drug dose-efficacy response surface and assess treatment efficacy for PRS Regimen II (Fig. 1b; Supplementary Table 2). Mice that were sham-treated had 6.71±0.05 log_10_ CFU of *M. tuberculosis* per lung at the end of the 4-week treatment period (Supplementary Table 2). Treatment with the Standard Regimen reduced lung burden of *M. tuberculosis* by 2.77 logs (*P*<0.0001, one-way ANOVA with Tukey's correction). Mice treated with any of the 10 dose variations of the PRS Regimen II (with various doses of EMB, BDQ and PZA) reduced bacterial burden in the lung by 4.75 to 5.95 logs to a level significantly lower than that achieved by the Standard Regimen (*P*<0.0001, one-way ANOVA with Tukey's correction). Mice in the best PRS Regimen II-treated group had 3.2 logs fewer CFU in their lung than mice treated with the Standard Regimen. Mice treated with PRS Regimen II also exhibited the least gross lung pathology and number of tubercles on the lung compared with mice that were sham-treated or treated with the Standard Regimen (Fig. 3). Thus, PRS Regimen II is significantly more efficacious than the Standard Regimen. PRS Regimen II is also more potent than PRS Regimen I, as indicated by a larger reduction in lung burden; indeed, in 4 out of 10 groups, no organism was detected in any of the animals. These two regimens differ by a single drug with PRO in PRS Regimen I replaced by BDQ in PRS Regimen II. With BDQ, instead of PRO in the regimen, variation of the PZA dose had a smaller impact on treatment efficacy (Fig. 1d, middle and bottom). However, decreasing the dose of BDQ alone or that of BDQ and PZA together adversely affected the efficacy of PRS Regimen II (Fig. 1d, bottom).

On the basis of lung log_10_ CFU data, a parabolic drug dose-efficacy response surface was generated for PRS Regimen II which had a fitting correlation of 0.995 and an adjusted *R*^2^ value of 0.981 (Methods, equation 3). A comparison of the observed means versus the predicted means of the response surface model showed a per cent difference of ≤6.8% with an average difference of 0.33%. One drug combination group (that is, HMMH) among the 10 PRS Regimen II groups was identified as an outlier under the model and was omitted in a repeat assessment. The model estimated that optimal doses of the drugs in the combination were about the same with or without this group in the model. Levene's test showed no deviation from homoscedasticity among 9 of the 10 PRS Regimen II s.d., satisfying the requirements for s.d. pooling in the parabolic and ANOVA analyses.

### PRS regimens rapidly render lungs culture negative

**Figure 4:**
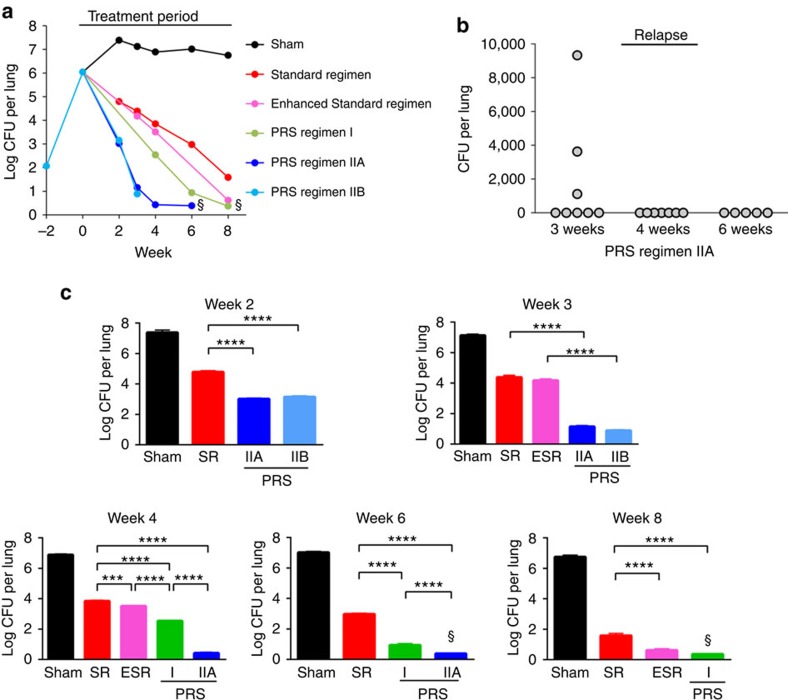
Medium-term efficacy and relapse study. (**a**) *M. tuberculosis* burden in the lung over the course of infection and treatment period, where mice were sham-treated or treated with the Standard Regimen (SR), Enhanced Standard Regimen (ESR) or PRS Regimen I, IIA or IIB starting at Week 0. Data are mean log_10_ CFU for *n*=5 mice per group. ^§^CFU at limit of detection. (**b**) Relapse 3 months after completion of treatment with PRS Regimen IIA for the duration indicated. (**c**) *M. tuberculosis* lung burden after treatment 5 days per week for 2, 3, 4, 6 and 8 weeks in sham-treated mice or mice treated with Standard Regimen (SR), Enhanced Standard Regimen (ESR) or PRS Regimen I, IIA or IIB. Data are mean±s.e.m. of log_10_ CFU for *n*=5 mice per group. Differences in efficacy between groups were evaluated by one-way ANOVA with Tukey's correction. ***P<0.001, ****P<0.0001. ^§^No *M. tuberculosis* CFU detected—data plotted as discussed in Methods.

To determine the treatment time required for the PRS regimens to render the lung of *M. tuberculosis-*infected mice culture negative, we conducted a direct comparison of the treatment efficacy of these two PRS regimens against the Standard Regimen (Tables 2 and 3). Since the lung burden of *M. tuberculosis* in PRS Regimen II-treated mice in the 4-week short-term study was at the experimental limit of detection when PZA was administered at both the low and high doses, we assessed PRS Regimen II in an 8-week treatment study with PZA both at the 150 mg kg^−1^ middle dose and the 450 mg kg^−1^ high dose, which we termed PRS Regimen IIA and IIB, respectively (Fig. 4; Table 1). In addition, we included in this study the Enhanced Standard Regimen, where the dose of PZA was increased to 450 mg kg^−1^, the same as in the optimized PRS Regimen I. Although the 150 mg kg^−1^ dose of PZA is commonly used in mouse studies^22^,^23^ and is thought to match the standard human dose, a higher 300 mg kg^−1^ dose of PZA has been shown to have greater efficacy in both mouse and guinea pig^24^ and the addition of a 450 mg kg^−1^ dose of PZA has been shown to increase the efficacy of a gatifloxacin, ethionamide combination in a mouse model of pulmonary TB (ref. 25).

**Figure 5:**
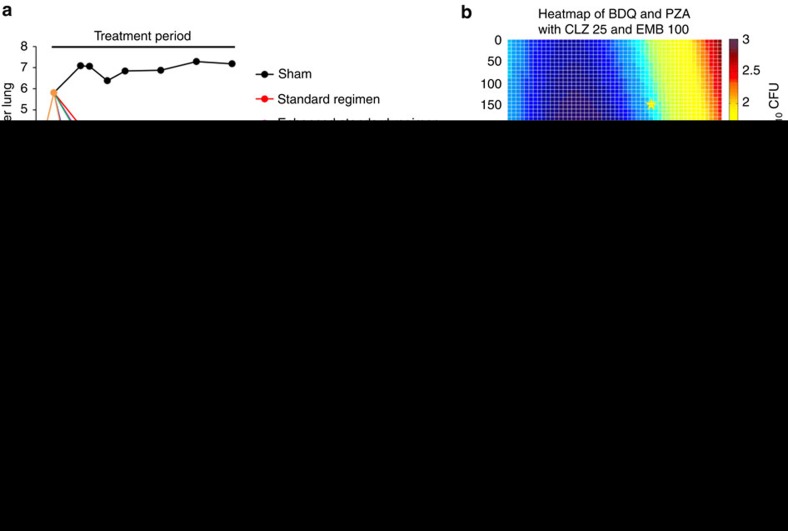
Long-term efficacy and relapse study. (**a**) *M. tuberculosis* burden in the lung over the course of infection and treatment period, where mice were sham-treated or treated with the Standard Regimen, Enhanced Standard Regimen or PRS Regimen I or IIC 5 days (Monday—Friday) a week starting at Week 0. The PRS Regimen IIC (daily) group was treated daily for 14 days. Data transformation as log_10_ (*x*+1) with *x* being the actual CFU was used for graphing purpose. (**b**) Heatmap for PRS Regimen II with CLZ and EMB dose at 25 and 100 mg kg^−1^, respectively, indicating that the optimal doses of BDQ and PZA were 30 and 450 mg kg^−1^, respectively. The white zone around BDQ 30 mg kg^−1^ at the bottom of the map corresponds to CFU projected to be 0 or below. (**c**) Relapse in the lung 3 months after completion of treatment with the PRS Regimen I or IIC or control regimens (Standard Regimen or Enhanced Standard Regimen) daily (d) or 5 days per week (wk) for the duration indicated. Differences in time to relapse-free cure between the Standard Regimen and the PRS regimens administered at the same frequency (5 days per week) were statistically significant (*P*=0.002 versus PRS Regimen I and *P*<0.0001 versus PRS Regimen IIC, log rank test). Differences between PRS Regimens I and II in time to relapse-free cure (administered at the same frequency (5 days per week)) were also statistically significant (*P*<0.0001, log rank test).

In this 8-week treatment study, at the time treatment was started two weeks post-challenge, the number of *M. tuberculosis* CFU per lung in infected mice was 6.04±0.08 log_10_ (Fig. 4a; Supplementary Table 3). Without treatment, *M. tuberculosis* continued to multiply, and by two weeks later, the lung burden of *M. tuberculosis* plateaued at ∼7 log_10_ CFU for the duration of the experiment. Treatment with the Standard Regimen reduced *M. tuberculosis* burden in the lung by 2.6 logs at week 2 and by 5.2 logs at week 8. Although the difference was not statistically significant at week 3, the Enhanced Standard Regimen was significantly more efficacious than the Standard Regimen at week 4 (*P*<0.001) and week 8 (*P*<0.0001, one-way ANOVA with Tukey's correction). At the end of the 8-week treatment study, *M. tuberculosis* could still be cultured from the lungs of mice treated with the Standard Regimen and Enhanced Standard Regimen. Indeed, in a subsequent 20-week long-term treatment study, we found that it took 16 weeks for the Standard Regimen and 12 weeks for Enhanced Standard Regimen to attain culture negativity in the lungs (Fig. 5a; Supplementary Table 4a).

As in the short-term study, bacterial load in the lungs of mice treated with PRS Regimen I was significantly less than that of the Standard Regimen at week 4 (*P*<0.0001, one-way ANOVA with Tukey's correction; Fig. 4a,c). Moreover, throughout the 8-week treatment period, lung CFU counts in mice treated with PRS Regimen I was lower than in mice treated with the Enhanced Standard Regimen; in both of these regimens, PZA was at the same dose of 450 mg kg^−1^. The time to attain lung culture negativity for mice treated with PRS Regimen I was 8 weeks, a much shorter duration than the 16 and 12 weeks of treatment required for the Standard Regimen and the Enhanced Standard Regimen, respectively (Fig. 5a; Supplementary Table 4a).

Both PRS Regimen IIA and IIB killed *M. tuberculosis* at a rate much faster than the Standard regimen, Enhanced Standard Regimen, and PRS Regimen I. While treatment with the Standard Regimen reduced lung burden of *M. tuberculosis* by 4 logs at week 6 and by 5 logs at week 8, treatment with PRS Regimen II achieved these levels at week 2 and 3, respectively (Fig. 4c). Thus, PRS Regimen II is superior to PRS Regimen I, and PRS Regimen I is significantly better than the Standard Regimen and the Enhanced Standard Regimen.

The drug dose-efficacy response surface generated for BDQ and PZA predicts a higher treatment efficacy for PRS Regimen II with BDQ at 30 mg kg^−1^ and PZA at 450 mg kg^−1^; this regimen was designated as PRS Regimen IIC (Fig. 5b; Table 1). Treatment with PRS Regimen IIC 5 days per week for merely 3 weeks reduced the number of *M. tuberculosis* to 1 organism per lung on average with 40% of the mice (2 out of 5 mice) culture negative in their lungs. Daily treatment for 3 weeks led to lung culture negativity in 80% of the mice (4 out of 5 mice) (Fig. 5a; Supplementary Table 4a).

### PRS regimens rapidly cure mice

To assess relapse after treatment, we held mice for an additional 3 months after the completion of their antibiotic treatment, at which time they were killed and their entire lungs and spleens plated for CFU (Supplementary Table 4b–f). Relapse was defined as the finding of 1 or more CFU in the lung or spleen; however, lung counts alone would have sufficed to define relapse as all relapsed animals had at least 1 CFU in the lung, and all of these mice had fewer or no CFU in the spleen. For the Standard Regimen, it took 16 weeks of treatment to clear the infection in the lungs and spleen and to achieve 100% relapse-free cure (Fig. 5c; Supplementary Table 4b). In contrast, for PRS Regimen I, relapse-free cure was achieved in 80% of mice (that is, 4 out of 5 mice) after 8 weeks treatment and 100% of mice after 12-weeks treatment (*P*=0.002 versus Standard Regimen, log rank test) (Fig. 5c; Supplementary Table 4d). Treatment with PRS Regimen IIA 5 days per week achieved relapse-free cure in 63% of mice at 3 weeks and 100% at 4 weeks (Fig. 4b and Supplementary Table 3). Treatment with PRS Regimen IIC 5 days per week achieved relapse-free cure in 80% of mice at 3 weeks and 100% at 4 weeks (Fig. 5c; Supplementary Table 4e). Treatment with PRS Regimen IIC daily further accelerated relapse-free cure, achieving relapse-free cure in 40% of mice by 2 weeks and 100% by three weeks (Fig. 5c; Supplementary Table 4f). Thus, compared with the Standard Regimen, PRS Regimen IIC, when administered at the same frequency (5 days per week), reduces the duration of treatment required to achieve 80% relapse-free cure from 12 weeks to 3 weeks and 100% relapse-free cure from 16 weeks to 4 weeks (*P*<0.0001 versus Standard Regimen, log rank test), a 75% reduction in time to relapse-free cure compared with the Standard Regimen. The difference in time to relapse-free cure between PRS Regimens I and IIC was highly statistically significant (*P*<0.0001, log rank test).

## Discussion

In this study, we employed PRS methodology to re-optimize the doses of two promising TB drug regimens *in vivo,* and we then evaluated the optimized regimens using a mouse model of pulmonary TB. Dose re-optimization by PRS methodology, as opposed to the conventional approach of dose scaling, can avoid failures that may occur in transitioning from *in vitro* to *in vivo* studies. Because PRS methodology is output driven, that is, only the phenotypic response—the final outcome of drug treatment (CFU of *M. tuberculosis* in the lung in this case)—is measured experimentally, it is agnostic to drug mechanism and such considerations as the metabolic state of the bacteria. Moreover, the experimentally calibrated drug dose-efficacy response surface automatically allows for drug–drug interactions; hence, pharmacokinetic studies are not necessary to determine the optimal drug doses. Thus, this approach describes the optimal drug doses for achieving the desired endpoint (maximum efficacy—reflected by the lowest log_10_ CFU in the lung in this case) without regard to the biological mechanisms that result in this outcome.

Both regimens comprised previously approved drugs and the first regimen (PRS Regimen I) comprised exclusively generic drugs. Both regimens were markedly more efficacious than the Standard Regimen in reducing the lung burden of *M. tuberculosis*. For example, at four weeks after the start of treatment, mice treated with PRS Regimen I had 1.3 logs fewer CFU in their lungs than mice treated with the Standard Regimen, while mice treated with PRS Regimen IIC had 4.0 logs fewer lung CFU counts than Standard Regimen-treated mice. Both regimens were also markedly more efficacious than the Standard Regimen in achieving relapse-free cure. Whereas the Standard Regimen required 12 weeks to achieve 80% relapse-free cure and 16 weeks to achieve 100% relapse-free cure in our *in vivo* model, PRS Regimen I achieved these milestones in 8 and 12 weeks, respectively, and PRS Regimen IIC in 3 and 4 weeks, respectively. Thus, compared with the Standard Regimen, PRS Regimens I and IIC reduced the duration of treatment required to achieve 80% relapse-free cure by 33 and 75%, respectively, and 100% relapse-free cure by 25% and 75%, respectively. In addition, as discussed below, PRS Regimen II achieves lung culture negativity and relapse-free cure in the mouse model faster than other regimens shown to accelerate treatment^26^,^27^,^28^,^29^. If our results in mice extrapolate to humans, PRS Regimen IIC would be expected to reduce the duration of treatment of TB from 6–8 months, as is currently the case for the Standard Regimen, to 1½–2 months. Moreover, as neither PRS Regimen I nor PRS Regimen II includes INH, RIF, fluoroquinolones or injectable aminoglycosides, these regimens should allow treatment, with a similar expedited time frame, of many cases of multidrug resistant TB (MDR-TB; defined as TB resistant to INH and RIF) and those cases of extensively drug-resistant TB (XDR-TB; defined as TB additionally resistant to fluoroquinolones and injectable aminoglycosides) in which susceptibility to PZA is preserved.

As others have done, we compared our PRS regimens to the Standard Regimen in the mouse model using the conventional mouse doses. While our PRS regimens were optimized in the mouse, the Standard Regimen was essentially optimized in humans and the mouse doses extrapolated from pharmacologic data. Nevertheless, the Standard Regimen extrapolated pharmacologically to mice is an important benchmark that is routinely used in mouse efficacy studies and provides an important reference point for comparisons between studies.

PRS Regimen II, in comparison with the Standard Regimen, reduces the time required to render the lung culture negative and achieve relapse-free cure by a greater amount (75%) than several previously reported regimens in the mouse model. For example, the combination of RIF/PZA/Moxifloxacin reduced the time to relapse-free cure by 33% (6 months to 4 months)^23^, and the combination of INH/PZA/RIF/CLZ by 50% (6 months to 3 months)^26^. Williams *et al*. included in their studies a drug combination of BDQ/PZA/Rifapentine/CLZ that is similar to our PRS Regimen II in that it shares 3 of the 4 drugs (they used Rifapentine instead of EMB; in addition, they used a PZA dose of 150 mg kg^−1^ rather than 450 mg kg^−1^) and observed relapse in 4/15 (27%) mice after 4 weeks treatment and relapse in 0/15 (0%) mice at 6 weeks^22^; the regimen was not compared with a standard regimen. In comparing even very similar regimens, however, it should be appreciated that because each drug regimen involves multiple potential positive and negative drug–drug interactions, that a change of just one drug in a multiple drug regimen may have a profound impact on efficacy. Rosenthal *et al*.^30^ observed that MXF/PZA (150 mg kg^−1^)/Rifapentine cleared bacteria in the lungs of mice after 2 months of treatment and reduced the time to relapse-free cure (0% relapse) by 50% compared with INH/PZA (150 mg kg^−1^)/RIF, which required 6 months to achieve relapse-free cure in all of the mice.

So far, our studies in the mouse TB model have explored only two of the drug combinations that our *in vitro* PRS study identified as being substantially more effective than the Standard Regimen, and in both cases our *in vivo* mouse studies confirmed the predictions of our *in vitro* macrophage studies. However, while antimicrobial efficacy in macrophages is important and probably a pre-requisite for efficacy *in vivo*, the properties of the drug combinations that make them highly effective *in vivo* may differ from the properties that make them effective in macrophage cell culture, and it is vitally important to test and optimize the drug combinations both *in vitro* and *in vivo*. For example, whereas the macrophage cell cultures are bathed in a constant concentration of antibiotics, it is likely that pharmacokinetics plays a major role in efficacy in the mouse model and that the very long half-lives of CLZ and BDQ make them potent components in the combination cocktails.

All TB drugs can occasionally cause severe adverse effects, some readily manageable and others not; even when not manageable, most adverse effects are reversible upon drug discontinuation. With respect to drug toxicity, the Standard Regimen comprises three hepatotoxic drugs (INH, RIF, PZA), three drugs that can cause gastrointestinal intolerance (INH, RIF, PZA), two drugs that can cause optic neuritis (EMB, INH) and a drug that can lead to peripheral neuropathy (INH), among other adverse effects^3^,^31^,^32^,^33^. Both PRS regimens share two of these drugs with the Standard Regimen (EMB, PZA) and also include CLZ, which can cause gastrointestinal intolerance, prolong the QTc interval, and result in reversible skin discoloration (bronzing)^33^. In addition, PRS Regimen I includes PRO, which can cause gastrointestinal intolerance and be hepatotoxic^33^, while PRS Regimen II includes BDQ, which can prolong the QTc interval and be hepatotoxic^34^. All things considered, managing patients on the PRS regimens should be no more difficult and probably less difficult than managing patients on the Standard Regimen.

One of the interesting findings in our PRS *in vitro* studies was that many of the most promising drug combinations included CLZ. Although the anti-TB activity of CLZ was identified in the 1950s, it has not featured prominently in TB treatment regimens because it was initially thought to be poorly active in patients with TB. However, interest in CLZ has resurfaced consequent to the challenge of treating MDR- and XDR-TB. A CLZ-containing regimen (combined with gatifloxacin, EMB, and PZA, and supplemented with PRO, kanamycin and high-dose INH for an intensive phase of at least 4 months) was shown both to be highly effective for treatment of MDR-TB and to allow duration of therapy for MDR-TB to be shortened from 20 months (or more) to 9–12 months^27^. Recent studies in a mouse model of TB have shown that addition of CLZ to a first-line regimen provides more rapid lung sterilization (3 versus 5 months) and shortens the duration of treatment needed to achieve relapse-free cure (3 versus 6 months)^26^. Similarly, addition of CLZ to a second-line drug regimen in a mouse model of INH-resistant TB also provided more effective lung sterilization and provided relapse-free cure after 9 months, whereas in the absence of CLZ, the mouse lung tissue was not sterilized after 9 months of treatment^28^. As monotherapy, CLZ does not show early bactericidal activity in mice^35^ or man^29^ and it did not contribute to early bactericidal activity in combination with other drugs in human studies^29^. Nevertheless, it is likely that CLZ is beneficial in combination regimens both because its long half-life provides sustained antibiotic coverage and because of synergistic activity with other antibiotics. From the standpoint of synergistic mechanisms, Williams *et al*. observed that PZA and CLZ increased the bactericidal activity of BDQ in their mouse model of pulmonary TB and hypothesized that BDQ, PZA, and CLZ may have additive effects through inhibition of ATP synthesis and that the ATP depleting activity of CLZ and BDQ could prevent the efflux of pyrazinoic acid^22^.

In PRS Regimen I, whereas PRO and EMB showed a relatively flat dose-response relationship, increasing the PZA dose yielded substantial increases in efficacy. A similar strong dose-response relationship was demonstrated by Ahmad *et al*.^24^ for PZA both as monotherapy and in combination with RIF, prompting these authors to suggest clinical studies to evaluate whether the clinically used dose of PZA should be increased. Although lower PZA doses of 150–300 mg kg^−1^ are more often used in mouse studies, we found that the 450 mg kg^−1^ dose of PZA was well tolerated by the mice. A surface area-based conversion factor (‘FDA Guidance for Industry: Estimating the Maximum Safe Starting Dose in Initial Clinical Trials for Therapeutics in Adult Healthy Volunteers', available at http://www.fda.gov/downloads/Drugs/.../Guidances/UCM078932.pdf) predicts the mouse 450 mg kg^−1^ dose to be equivalent to a 36.6 mg kg^−1^ dose for a 60 kg human, and this dose is only slightly higher than the clinically recommended dose^27^, just above the upper end of the 15–30 mg kg^−1^ dosing range recommended by the CDC-ATS guidelines^36^. Comparison of published PZA PK data in mice and humans also suggest that the standard 150 mg kg^−1^ dose of PZA in mice yields a lower AUC than a human dose of 30 mg kg^−1^. In humans, an oral PZA dose of 27 mg kg^−1^ yielded an AUC of 520 mg h l^−1^ in one study^37^ and in another study a dose of 22–30 mg kg^−1^ yielded an AUC of 617–738 mg h l^−1^ (ref. 38). In contrast, an oral PZA dose of 150 mg kg^−1^ in BALB/c mice yielded significantly lower AUCs of 350 mg h l^−1^ (ref. 39) and 388 mg h l^−1^ (ref. 38). Moreover, PZA doses of 50–60 mg kg^−1^ have been used clinically and a systematic review and meta-analysis determined that higher doses of PZA were not associated with any dose-dependent increase in hepatotoxicity^40^. Indeed, the concerns over PZA related hepatotoxicity have been in the setting in which it is used in the Standard Regimen in combination with the hepatotoxic drugs INH and RIF; thus, the Enhanced Standard Regimen tested in our mouse studies, where the PZA dose was substantially increased compared with the pharmacologically equivalent human dose and which showed improved efficacy compared with the Standard Regimen in the mouse, is not likely to extrapolate safely to humans. In contrast, PRS Regimens I and II do not include these additional two hepatotoxic drugs, making it possible to safely administer a substantially higher dose of PZA. While higher doses of PZA in the setting of PRS Regimens I and II could be associated with an increase in other side effects such as arthralgias and elevated uric acid levels, these are likely to be manageable and the shorter course of therapy would likely reduce the incidence of serious adverse events. Of note, however, while the higher dose of PZA may be ideal, for both PRS Regimens I and II, our drug dose-efficacy response surface model indicates that lower doses of PZA can be used without significantly compromising efficacy.

Our previous *in vitro* PRS studies employed PRO rather than ethionamide (ETA) because, although the two drugs are considered interchangeable, PRO has been found to outperform ETA in a clinical trial of leprosy and to be better tolerated than ETA (ref. 33). Nevertheless, we anticipate that ETA could replace PRO in our PRS regimens without loss of efficacy.

PRS I Regimen I (CLZ/EMB/PRO/PZA) has the advantage of being inexpensive and these drugs are readily available on the formulary in most countries. On the basis of our mouse studies, we predict that PRS Regimen II (CLZ/EMB/BDQ/PZA) will provide even more rapid bacterial clearance in the tissues and will achieve relapse-free cure even sooner.

Whether our mouse model will accurately predict the results in humans remains to be determined. There are reasons why the model may overestimate or underestimate the efficacy of various drug combinations. As others have noted^22^, some differences between TB in mouse and human could lead the BALB/c model to overestimate the clinical efficacy of BDQ and CLZ. For example, unlike humans, the BALB/c mouse does not develop cavitary disease and the bacteria are almost exclusively intracellular. Since CLZ and BDQ are very hydrophobic, they become concentrated intracellularly and might be less effective against extracellular TB (ref. 22). Consistent with this, Irwin *et al*.^41^ recently observed that CLZ showed much greater TB killing activity in BALB/c mice than in C3HeB/FeJ mice that develop caseous necrosis with numerous extracellular TB. It is possible that the duration of treatment may require adjustment depending on the presence or absence of cavitary disease. On the other hand, the mouse, which does not develop as strong a cell-mediated immune response as humans, as reflected by less organized granulomata and relatively weak cutaneous delayed-type hypersensitivity responses^42^, may underestimate the clinical efficacy of drugs at achieving relapse-free cure. Our studies indicate that as few as 1 or 2 bacteria remaining in the lung at the cessation of treatment are sufficient to allow relapse in our mouse model.

Our studies demonstrate that systematic *in vitro* and *in vivo* PRS methodology can identify and optimize drug combinations for treatment of TB that are substantially more effective than the current Standard Regimen. Expanding the PRS methodology to additional drug combinations, including some of the newly developed drugs, has the potential to identify combinations that are even more potent.

## Methods

### Aerosol infection

Eight-week old, female, pathogen-free BALB/c mice were obtained from Taconic, housed in groups of five, and provided with unlimited access to food and water. Mice were challenged by aerosol with the highly virulent *M. tuberculosis* Erdman strain (ATCC 35801). One day later, two mice were killed to determine the initial number of CFU implanted in their lungs. After two weeks, three mice were killed to determine the number of CFU in the lungs at the start of treatment.

### Drug treatment

Two weeks after infection, mice were treated by oral gavage five times per week (Monday—Friday) for 3–24 weeks, or sometimes daily for 2–3 weeks. Mice under treatment with the Standard Regimen were first administered EMB, INH, and PZA together in 0.1 ml of 0.15% agarose suspension, and 1 h later administered RIF in water. Mice under treatment with PRS regimens were first administered CLZ alone in 0.1 ml of 0.15% agarose suspension and 1 h later administered EMB/PRO/PZA (PRS Regimen I) or EMB/BDQ/PZA (PRS Regimen II) together in 0.1 ml 0.15% agarose suspension. Sham-treated mice were first administered agarose suspension and 1 h later administered water. CLZ, EMB, INH, PRO, RIF and PZA were purchased from Sigma-Aldrich. BDQ was provided by the Global Alliance for TB Drug Development. Drug doses used in this study are listed in Table 1.

### Assessment of treatment bactericidal activity

Mice were killed three days after the last treatment dose. Their lung, liver and spleen were homogenized in 1 ml of phosphate-buffered saline. The homogenates were serially diluted and spread 100 μl per plate on 7H11 agar containing 0.4% activated charcoal, ampicillin (12.5 μg ml^−1^), amphotericin (5 μg ml^−1^) and polymyxin B (20 U ml^−1^). The plates were incubated at 37 °C in a 5% CO_2_–95% air atmosphere for 31 days, after which the number of CFU of *M. tuberculosis* on each plate was enumerated. The limit of detection for CFU ranged from 4.4 to 5 organisms per lung depending on the total volume of lung homogenate from each animal. When no organism was detected on any of the plates, the CFU per lung was plotted as half of the limit of detection. When only a single colony was detected on all of the plates, the CFU per lung was plotted as the limit of detection.

### Assessment of relapse

Mice were held for 3 months after the last treatment; killed; the lung and spleen homogenized; and the entire homogenate plated on 60 (20 for each lung and 40 for each spleen) charcoal 7H11 agar plates. CFU on the plates was determined after incubation for 31 days at 37 °C in a 5% CO_2_–95% air atmosphere. The absence of a single CFU in the entire lungs of mice 3 months after treatment cessation was considered relapse-free cure.

### Animal studies

All animal studies were approved and conducted in accordance with the procedures as set forth by the UCLA Animal Research Committee. The BALB/c mouse strain was used as this is the established model for studies of the efficacy of multidrug TB regimens^10^,^22^,^23^,^26^,^28^,^30^,^35^,^43^,^44^,^45^,^46^. Experience has shown that the results of multidrug efficacy studies in the mouse model of pulmonary tuberculosis are generally, albeit not perfectly, predictive of their efficacy in humans^10^,^11^,^12^.

### Parabolic drug dose-efficacy response surface

In a previous study^8^, we identified highly promising drug combinations for killing *M. tuberculosis* in human macrophages. Here, we evaluate two of these combinations (PRS Regimens I and II), each consisting of 4 drugs, *in vivo* in the mouse model of pulmonary tuberculosis. To transition from *in vitro* to *in vivo* studies, we first needed to identify the optimal doses of these drugs in each PRS regimen *in vivo.* This was accomplished using PRS methodology^8^, which in essence describes the relationship between a phenotypic response in animals (the output measurement, in this case the lung burden of *M. tuberculosis*) and doses of different drugs used to effect this phenotypic response. Based upon our previous findings in multiple complex biological settings, including i*n vitro* studies involving eukaryotic cells, *in vivo* studies, and clinical studies^17^,^18^,^19^,^47^,^48^,^49^, the relationship between the phenotypic response and the doses of drugs used to effect the phenotypic response fits a parabolic response surface (sometimes referred to herein as the drug dose-efficacy response surface). Such a surface can be described by a second order algebraic (quadratic) equation (see below), as opposed to much more complex surfaces requiring, for example, third and fourth order algebraic equations to describe them. A major advantage of the PRS approach is that a relatively small number of tests is required to determine the coefficients of the second order algebraic equation and thus define the parabolic response surface. In the current study, the drug combinations each contained 4 drugs; ordinarily the second order algebraic equation for four drugs would have 15 coefficients, which can be determined by 15 tests (or in this case groups of animals receiving different drug-dose combinations). By keeping the dose of one drug (CLZ) in each combination constant, we reduced the number of tests required to 10, the number of coefficients in the second order algebraic equation for 3 drugs; this allowed substantial savings in cost, labour, and time. Thus, only 10 groups of animals, where each group was treated with a different dose ratio of the three drugs, were needed to map out the drug-dose efficacy response surface. We chose CLZ as the drug to be kept at a constant dose because CLZ has an extraordinarily long half-life^35^. Moreover, Swanson *et al*. have recently shown in a mouse model of TB that CLZ exhibits a threshold effect, such that differences in CLZ concentrations in the blood and tissues do not translate into dose-dependent differences in antimicrobial activity^35^.

Specifically, in this study, the lung burden of *M. tuberculosis* (log_10_ CFU) in mice after treatment with PRS Regimen I or II drugs at various drug doses was used to generate the second-order (parabolic) algebraic model using MATLAB LinearModel.stepwise function (Mathworks Inc., Natick, MA); and the correlation between experimental and modelled log_10_ CFU was determined using the MATLAB linear or rank correlation function. The parabolic response surface is expressed as:


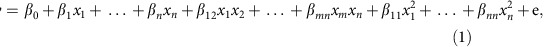


where *y* represents the log CFU; *x*_*n*_ is the *n*th drug dosage; *β*_0_ is the intercept term; *β*_*n*_ is the single drug coefficient of the *n*th drug; *β*_*mn*_ is the interaction coefficient between the *m*th and *n*th drugs; *β*_*nn*_ is the quadratic coefficient for the *n*th drug; and e is the residual error assumed to have a normal distribution with constant s.d.=σ_e_.

As noted above, in determining the parabolic response surface, by keeping the dose of one drug in the combination constant, we reduced the number of unknown coefficients (*β*s) in equation (1) above to 10. Therefore, in this study, *n*=3 in equation (1). We applied the Orthogonal Array Composite Designs (OACD) method to place the 10 drug-dose combinations (experimental groups) at strategic positions for locating the most accurate parabolic response surface^15^,^16^. This was done by selecting high (H), medium (M) and low (L) drug doses of each drug and ensuring that each pair of conditions (for example, HH, HM, HL, MH, MM, ML, LH, LM and LL) for any two drugs occurred equally often for all of the drug pairs in the combinations being tested. The use of an orthogonal array test design to map the parabolic response surface allowed us to maximize our coverage of the drug dose-efficacy response surface, while minimizing the number of combinations to be tested.

A parabolic regression model was fit to the log_10_ CFU data using equation (1) above, giving the expected log_10_ CFU as a function of the drug dose combinations. For every observed log_10_ CFU, a corresponding predicted log_10_ CFU value was generated by this equation. The residual errors (e) were defined as the difference between the observed log_10_ CFU and the predicted log_10_ CFU (e=observed log_10_ CFU—predicted log_10_ CFU). A normal quantile plot of the residual errors confirmed that they followed a normal distribution.

In determining the final parabolic response surface, we removed non-significant terms from the original 10 term equation using a stepwise hierarchical removal rule^50^ and a *p* <0.05 term retention criterion. For PRS Regimen I, the parabolic drug dose-efficacy response surface determined from the lung log_10_ CFU data is given in equation (2), below:


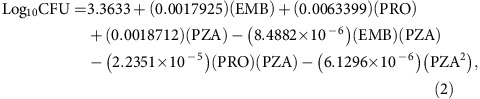


where EMB, PRO and PZA represent the dose of EMB, PRO and PZA, respectively, in mg kg^−1^.

For PRS Regimen II, the parabolic drug dose-efficacy response surface determined from the lung log_10_ CFU data is given in equation (3), below:


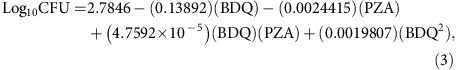


where BDQ and PZA represent the dose of BDQ and PZA, respectively, in mg kg^−1^.

Once the parabolic drug dose-efficacy response surface was obtained from the experimental data, the optimal drug doses were derived as those giving the lowest projected mean number of *M. tuberculosis* (log_10_ CFU) in the mouse lung.

### Statistics

On the basis of previous studies^10^,^28^ showing that mean differences in log_10_ CFU were 2 s.d. or larger, we chose a sample size of 5 mice per group as it provided 80% power to confirm mean differences of 2 s.d. or larger using the *P*<0.05 significance criterion. Per cent relapse versus treatment time were compared across groups using the log rank test since this data did not follow the normal distribution and most observations were censored (that is the animal did not relapse). Means and s.e. of the mean (s.e.m.) are reported. Data collected from the animal studies were analysed using GraphPad Prism software (version 6.05). Means were compared across groups by ANOVA with Tukey's correction for multiple comparisons. Levene's test was used to assess error homoscedasticity for both the parabolic model and the ANOVA.

Additional statistical analyses of the parabolic drug dose-efficacy response surface model are described in the preceding section entitled Parabolic drug dose-efficacy response surface.

### Code availability

The functions used for parabolic curve fitting to obtain the coefficients of the second order algebraic equation and for statistical analysis are available in MATLAB. An example of the MATLAB code used for parabolic curve fitting so as to obtain the coefficients of the second order algebraic equation and for statistical analysis is provided as Supplementary Software.

### Data availability

All data generated or analysed during this study are included in this published article and its supplementary information files, or are available from the corresponding author on request.

## Additional information

**How to cite this article**: Lee, B.-Y. *et al*. Drug regimens identified and optimized by output-driven platform markedly reduce tuberculosis treatment time. *Nat. Commun.* **8**, 14183 doi: 10.1038/ncomms14183 (2017).

**Publisher's note**: Springer Nature remains neutral with regard to jurisdictional claims in published maps and institutional affiliations.

## Supplementary Material

Supplementary Information Supplementary Tables

Supplementary Software Example of MATLAB code used for parabolic curve fitting to obtain the coefficients and statistics of the second order algebraic equation.

## Figures and Tables

**Table 1:** Drugs and drug doses used in the studies.

Treatment	Drug dose (mg kg^−1^)			
Control Standard Regimen	INH	RIF	EMB	PZA
Standard Regimen	25	10	100	150
Enhanced Standard Regimen	25	10	100	450
				
Experimental TB Drug Regimens	CLZ	EMB	PRO	PZA
PRS Regimen I				
H/L/L/H	25	11.1	8.3	450
H/L/H/L	25	11.1	75	50
H/H/L/L	25	100	8.3	50
H/H/H/H	25	100	75	450
H/H/H/L	25	100	75	50
H/H/L/H	25	100	8.3	450
H/L/H/H	25	11.1	75	450
H/H/M/M	25	100	25	150
H/M/H/M	25	33.3	75	150
H/M/M/H	25	33.3	25	450
				
	CLZ	EMB	BDQ	PZA
PRS Regimen II				
H/L/L/H	25	11.1	5.6	450
H/L/H/L	25	11.1	50	50
H/H/L/L	25	100	5.6	50
H/H/H/H	25	100	50	450
H/H/H/L	25	100	50	50
H/H/L/H	25	100	5.6	450
H/L/H/H	25	11.1	50	450
H/H/M/M	25	100	16.7	150
H/M/H/M	25	33.3	50	150
H/M/M/H	25	33.3	16.7	450
PRS Regimen IIA	25	100	16.7	150
PRS Regimen IIB	25	100	16.7	450
PRS Regimen IIC	25	100	30	450

**Table 2:** Scheme of medium-term efficacy and relapse study.

**Treatment**†	**Mice**	**Week**						
		(Number of mice killed per group)*						
		**−2**	**0**	**2**	**3**	**4**	**6**	**8**
Untreated	7	2	5					
Sham	25			5	5	5	5	5
Standard Regimen	25			5	5	5	5	5
Enhanced Standard Regimen	15				5	5		5
PRS Regimen I	15					5	5	5
PRS Regimen IIA	40			5	5 8‡	5 7‡	5 5‡	
PRS Regimen IIB	10			5	5			

^*^Number of mice killed per group at 3 days (Efficacy) or 3 months (Relapse) after completion of treatment for the period indicated to determine lung burden of *M. tuberculosis*.

^†^Mice were treated starting two weeks after infection by oral gavage, 5 days per week.

^‡^Number of mice held for 3 months after cessation of treatment for the time indicated for assessment of relapse. The availability of five extra mice allowed the Week 3 and Week 4 groups to be increased in size by 3 and 2 mice, respectively.

**Table 3:** Scheme of long-term efficacy and relapse study.

**Treatment**†	**Mice**	**Week**									
		(Number of mice killed per group)*									
		**−2**	**0**	**2**	**3**	**4**	**6**	**8**	**12**	**16**	**20**
Untreated	8	3	5								
Sham	35				5	5	5	5	5	5	5
Standard Regimen	50				5	5	5	5	5 5‡	5 5‡	5 5‡
Enhanced Standard Regimen	50				5	5	5	5	5 5‡	5 5‡	5 5‡
PRS Regimen I	20							5 5‡	5 5‡		
PRS Regimen IIC	30				5 5‡	5 5‡	5 5‡				
PRS Regimen IIC daily	20			5 5‡	5 5‡						

^*^Number of mice killed per group 3 days (Efficacy) or 3 months (Relapse) after completion of treatment for the time indicated to determine lung burden of *M. tuberculosis*.

^†^Starting 2 weeks after challenge, mice were treated by oral gavage 5 days per week or, in the case of one group treated with PRS Regimen IIC, daily, as indicated in the bottom row. Mice treated with the Standard Regimen or Enhanced Standard Regimen were administered RIF and EMB/INH/PZA by gavage for up to 8 weeks, and thereafter administered RIF and INH alone.

^‡^Number of mice held for 3 months after completion of treatment for the time indicated for assessment of relapse.
