# Supplementary material for: Drug regimens identified and optimized by output-driven platform markedly reduce tuberculosis treatment time
Source: Nat Commun. 2017 Jan 24;8:14183. doi: 10.1038/ncomms14183 (PMC5287291; doi:10.1038/ncomms14183)
Supplement: Supplementary Information — Supplementary Tables [file ncomms14183-s1.pdf]

Supplementary Table 1. Lung burden of *M. tuberculosis* in PRS Regimen I short-term efficacy study

Group	Treatment	Drug	Dose†	Log$_{10}$ CFU (Mean ± s.e.m.)^		
				Day 1	Day 14	Day 42
A	Sham	None		2.22 ± 0.09	6.24 ± 0.05	6.67 ± 0.09
B	Standard Regimen	INH/RIF/EMB/PZA				4.24 ± 0.08
C	PRS Regimen I	CLZ/EMB/PRO/PZA	H/L/L/H			2.88 ± 0.11
D	PRS Regimen I	CLZ/EMB/PRO/PZA	H/L/H/L			3.83 ± 0.16
E	PRS Regimen I	CLZ/EMB/PRO/PZA	H/H/L/L			3.64 ± 0.10
F	PRS Regimen I	CLZ/EMB/PRO/PZA	H/H/H/H			2.45 ± 0.11
G	PRS Regimen I	CLZ/EMB/PRO/PZA	H/H/H/L			3.97 ± 0.09
H	PRS Regimen I	CLZ/EMB/PRO/PZA	H/H/L/H			2.77 ± 0.07
I	PRS Regimen I	CLZ/EMB/PRO/PZA	H/L/H/H			2.69 ± 0.07
J	PRS Regimen I	CLZ/EMB/PRO/PZA	H/H/M/M			3.60 ± 0.10
K	PRS Regimen I	CLZ/EMB/PRO/PZA	H/M/H/M			3.78 ± 0.06
L	PRS Regimen I	CLZ/EMB/PRO/PZA	H/M/M/H			2.80 ± 0.05

† Drug doses for the Standard Regimen and PRS Regimen II are shown in Table 1.

^Mice were infected with *M. tuberculosis* by aerosol at day 0, and to monitor bacterial growth during the pretreatment period, several mice were euthanized at day 1 and day 14 for determination of *M. tuberculosis* CFU counts in the lung. Starting at day 14, the mice were treated 5 days (Monday – Friday) per week for 4 weeks, and euthanized at day 42, 3 days after the last treatment dose, for determination of lung CFU counts.

Supplementary Table 2. Lung burden of *M. tuberculosis* in the PRS Regimen II short-term efficacy study

				Log_{10}CFU (Mean ± s.e.m.)^§		
Group	Treatment	Drug	Dose†	Day 1	Day 14	Day 42
A	Sham	None		2.36 ± 0.11	6.09 ± 0.04	6.71 ± 0.05
B	Standard Regimen	INH/RIF/EMB/PZA				3.94 ± 0.03
C	PRS Regimen II	CLZ/EMB/BDQ/PZA	H/L/L/H			1.02 ± 0.12
D	PRS Regimen II	CLZ/EMB/BDQ/PZA	H/L/H/L			0.64 ± 0.12
E	PRS Regimen II	CLZ/EMB/BDQ/PZA	H/H/L/L			1.96 ± 0.09
F	PRS Regimen II	CLZ/EMB/BDQ/PZA	H/H/H/H			0.46 ± 0.01§
G	PRS Regimen II	CLZ/EMB/BDQ/PZA	H/H/H/L			0.47 ± 0.00§
H	PRS Regimen II	CLZ/EMB/BDQ/PZA	H/H/L/H			1.10 ± 0.19
I	PRS Regimen II	CLZ/EMB/BDQ/PZA	H/L/H/H			0.52 ± 0.06§
J	PRS Regimen II	CLZ/EMB/BDQ/PZA	H/H/M/M			0.47 ± 0.01§
K	PRS Regimen II	CLZ/EMB/BDQ/PZA	H/M/H/M			0.45 ± 0.01§
L	PRS Regimen II	CLZ/EMB/BDQ/PZA	H/M/M/H			0.54 ± 0.07

†Drug doses for Standard Regimen and PRS Regimen II are shown in table S1.

^Mice were infected with *M. tuberculosis* by aerosol at day 0, and to monitor bacterial growth during the pretreatment period, several mice were euthanized at day 1 and day 14 for determination of *M. tuberculosis* CFU counts in the lung. Starting at day 14, the mice were treated 5 days (Monday – Friday) per week for 4 weeks, and euthanized at day 42, 3 days after the last treatment dose, for determination of lung CFU counts.

§No *M. tuberculosis* CFU were detected on plates - data stated as discussed in methods

Supplementary Table 3. Organ burden of *M. tuberculosis* in medium-term study

a. Efficacy†

Treatment Week	Sham	Standard Regimen	Enhanced Standard Regimen	PRS Regimen I	PRS Regimen IIA	PRS Regimen IIB
-2	2.07 ± 0.08					
0	6.04 ± 0.08					
2	7.39 ± 0.16	4.79 ± 0.08			3.02 ± 0.06	3.15 ± 0.08
3	7.12 ± 0.10	4.39 ± 0.13	4.18 ± 0.10		1.15 ± 0.06	0.89 ± 0.07
4	6.89 ± 0.05	3.85 ± 0.06	3.51 ± 0.02	2.54 ± 0.04	0.43 ± 0.06	
6	7.01 ± 0.08	2.97 ± 0.06		0.94 ± 0.11	0.39 ± 0.02	
8	6.75 ± 0.12	1.58 ± 0.15	0.62 ± 0.12	0.37 ± 0.01		

†Data shown are the mean ± s.e.m. Log_{10} CFU of *M. tuberculosis* burden in the lung.

b. Relapse†

	Lung			Spleen		
Mouse^	3 week	4 week	6 week	3 week	4 week	6 week
1	3635	0	0	127	0	0
2	9333	0	0	0	0	0
3	1120	0	0	0	0	0
4	0	0	0	0	0	0
5	1	0	0	0	0	0
6	0	0		0	0	
7	0	0		0	0	
8	0			0		

†Data shown are *M. tuberculosis* CFU counts in the whole organ 3 months after completion of treatment with PRS Regimen IIA for the indicated duration.

^The number of mice included in the 3-, 4-, and 6-week relapse study was 8, 7, and 5, respectively.

Supplementary Table 4. Lung burden of *M. tuberculosis* in long-term efficacy and relapse study

a. Efficacy†

Treatment Week	Sham	Standard Regimen	Enhanced Standard Regimen	PRS Regimen I	PRS Regimen IIC	PRS Regimen IIC (daily)
	Log_{10} CFU	Log_{10} CFU [Total CFU]	Log_{10} CFU [Total CFU]	[Total CFU]	[Total CFU]	Log_{10} CFU [Total CFU]
-2	2.30 ± 0.03					
0	5.68 ± 0.21					
2						1.70 ± 0.10
3	7.06 ± 0.07	4.21 ± 0.06	3.75 ± 0.07		[1.0 ± 0.4]	[0.2 ± 0.2]
4	7.04 ± 0.07	4.00 ± 0.03	2.86 ± 0.08		[1.0 ± 0.4]	
6	6.36 ± 0.07	2.87 ± 0.07	1.44 ± 0.07		[0.4 ± 0.2]	
8	6.75 ± 0.14	1.45 ± 0.05	[0.8 ± 0.2]	[0.0 ± 0.0]		
12	6.83 ± 0.09	[0.2 ± 0.2]	[0.0 ± 0.0]*	[0.0 ± 0.0]‡		
16	7.09 ± 0.19	[0.0 ± 0.0]	[0.0 ± 0.0]			
20	7.03 ± 0.17	[0.0 ± 0.0]	[0.0 ± 0.0]			
24	7.05 ± 0.11	[13.2 ± 13.2]	[0.0 ± 0.0]#			

†Data shown are *M. tuberculosis* CFU counts in the entire organ, either as Log_{10} CFU, or if in brackets, as Total CFU counts (Mean ± s.e.m.). Mice were treated starting two weeks after infection by oral gavage 5 days per week or, in the case of one group of mice on PRS Regimen IIC, daily as indicated in the last column.

*15% of plates were contaminated with a fungus

# Represents data from 4 mice; 20% of plates were contaminated with a bacterium

‡Represents data from 4 mice; 46% of plates were contaminated with a fungus

b. Relapse for Standard Regimen treatment 5 days per week†

	Lung			Spleen		
Mouse	12 weeks	16 weeks	20 weeks	12 weeks	16 weeks	20 weeks
1	0	0	0	0	0	0
2	0	0	0	0	0	0
3	0	0	0	0	0	0
4	0	0	0	0	0	0
5	323	0	0	0	0	0

†Data shown are total *M. tuberculosis* CFU counts for the whole organ 3 months after completion of treatment with the Standard Regimen for the indicated duration.

c. Relapse for Enhanced Standard Regimen treatment 5 days per week†

	Lung			Spleen		
Mouse	12 weeks	16 weeks	20 weeks	12 weeks	16 weeks	20 weeks
1	0	0	0	0	0	0
2	0	0	0	0	0	0
3	0	0	0	0	0	0
4	0	0	0	0	0	0
5	0	0	0	0	0	0

†Data shown are *M. tuberculosis* CFU counts from the whole organ 3 months after completion of treatment with the Enhanced Standard Regimen for the indicated duration.

d. Relapse for PRS Regimen I treatment 5 days per week†

	Lung		Spleen	
Mouse	8 weeks	12 weeks	8 weeks	12 weeks
1	0	0	0	0
2	0	0	0	0
3	0	0	0	0
4	0	0	0	0
5	453	0	0	0

†Data shown are *M. tuberculosis* CFU counts from the whole organ 3months after completion of treatment with PRS Regimen I for the indicated duration.

e. Relapse for PRS Regimen IIC treatment 5 days per week†

	Lung			Spleen		
Mouse	3 weeks	4 weeks	6 weeks	3 weeks	4 weeks	6 weeks
1	0	0	0	0	0	0
2	0	0	0	0	0	0
3	1164	0	0	0	0	0
4	0	0	0	0	0	0
5	0	0	47	0	0	0

†Data shown are *M. tuberculosis* CFU counts from the whole organ 3 months after completion of treatment with PRS Regimen IIC for the indicated duration.

f. Relapse for PRS Regimen IIC daily treatment†

	Lung		Spleen	
Mouse	14 days	21 days	14 days	21 days
1	1568	0	6	0
2	140	0	0	0
3	1521	0	114	0
4	0	0	0	0
5	0	0	0	0

†Data shown are *M. tuberculosis* CFU counts from the whole organ 3 months after completion of treatment with PRS Regimen IIC for the indicated duration.
